# Are we missing hypoglycaemia? Elderly patients with insulin-treated diabetes present to primary care frequently with non-specific symptoms associated with hypoglycaemia

**DOI:** 10.1016/j.pcd.2017.08.004

**Published:** 2018-04

**Authors:** Suzy V. Hope, Phil J. Taylor, Beverley M. Shields, Andrew T. Hattersley, Willie Hamilton

**Affiliations:** a Exeter NIHR Clinical Research Facility, RILD Building, Royal Devon & Exeter Hospital, Barrack Road, Exeter, Devon EX2 5DW, UK; b Axminster Medical Practice, St Thomas Court, Church Street, Axminster, EX13 5AG Devon, UK; c Department of Primary Care, University of Exeter Medical School, St Luke’s Campus, University of Exeter, Heavitree Rd, Exeter, Devon EX1 2LU, UK

**Keywords:** Elderly, Hypoglycaemia, Diagnosis, Symptoms, Insulin, Falls

## Abstract

**Introduction:**

We assessed if patients with known hypoglycaemia present on other occasions with non-specific symptoms associated with (but not diagnosed as) hypoglycaemia, potentially representing missed hypoglycaemia.

**Methods:**

335 primary care records (5/2/12-4/2/13) from patients aged >65 (79 on insulin, 85 on sulphonylureas, 121 on metformin only, 50 without diabetes) were assessed for hypoglycaemia episodes and consultations with non-specific symptoms, “hypo clues”.

**Results:**

27/79(34%) insulin-treated patients had >1 documented hypoglycaemia episode, compared to 4/85(5%) sulphonylurea-treated patients, 2/121(2%) metformin-only treated patients, and none without diabetes, p < 0.001.

“Hypo clue” consultations were common: 1.37 consultations/patient/year in insulin-treated patients, 0.98/patient/year in sulphonylurea-treated, 0.97/patient/year in metformin only-treated, and 0.78/patient/year in non-diabetic patients, p = 0.34. In insulin-treated patients with documented hypoglycaemia, 20/27(74%) attended on another occasion with a “hypo clue” symptom, compared to 21/52(40%) of those without hypoglycaemia, p = 0.008. No significant difference in the other treatment groups.

Nausea, falls and unsteadiness were the most discriminatory symptoms: 7/33(21%) with hypoglycaemia attended on another occasion with nausea compared to 14/302(5%) without hypoglycaemia, p = 0.002; 10/33(30%) vs 36/302(12%) with falls, p = 0.007; and 5/33(15%) vs 13/302(4%) with unsteadiness, p = 0.023.

**Conclusions:**

Non-specific symptoms are common in those >65 years. In insulin-treated patients at high hypoglycaemia risk, nausea, falls and unsteadiness should prompt consideration of hypoglycaemia.

## Highlights

• Non-specific symptoms make hypoglycaemia recognition difficult in older patients
• Patients aged >65 commonly present to primary care with non-specific symptoms
• Those with documented hypos present more on other occasions with non-specific symptoms, which could represent unrecognised hypoglycaemia
• Nausea, falls & unsteadiness were more common in these patients.

## 1 Introduction

Tight glycaemic control in order to prevent long-term complications of diabetes [1], [2] has been associated with an increased prevalence of hypoglycaemia in Type 1 [3] and Type 2 diabetes [4]. Increasing prevalence of diabetes coupled with longer life expectancy, and thus longer diabetes duration, means there is an increasing elderly population on potentially hypoglycaemia-causing medications such as insulin and sulphonylureas [5], [6], [7].

Hypoglycaemia has numerous direct risks such as falls, accidents, hospitalisation, impact on driving, fear and adverse effects on quality of life, arrhythmias, and long-term cognition [8]. It also brings the risk of becoming less aware of the symptoms – hypoglycaemia unawareness – which leads into a vicious circle [9].

The recognition that elderly people on hypoglycaemia-causing medications may be particularly vulnerable has led to alterations of guidelines, incorporating more relaxed HbA1c targets for frail elderly, or those with multiple comorbidities [10], [11], [12].

Hypoglycaemia symptoms in elderly people are less pronounced than in younger patients [13], [14], [15]. Hypoglycaemia is under-reported, and under-recognised – by patients, carers and healthcare professionals [16], [17], [18]. Symptoms also vary much more between episodes in the same person than is often appreciated [19]. These factors complicate estimates of the prevalence of hypoglycaemia, almost certainly leading to under-estimation.

As blood sugar levels fall, the autonomic symptoms of sweating, palpitations, and anxiety first occur; these stimulate food intake, in order to restore blood glucose levels [20]. However autonomic symptoms become less prominent in older age [13], [21], and glucose levels may thus fall into the “neuroglycopenic” range before self-correction. Symptoms of insufficient cerebral glucose are non-specific, including unsteadiness, light-headedness, tiredness and confusion [8], [22] – symptoms seen commonly in the general population [23], [24], [25], and particularly in elderly patients for many other reasons too [26], [27].

The symptoms most associated with hypoglycaemia have been reported [9], [20], [28], [29], including those particularly seen in the elderly [14]. However, their non-specific nature, along with multiple alternative explanations, including possible co-morbidities, mean that hypoglycaemia may not be recognised. This study aimed to establish if patients at risk of hypoglycaemia present more to primary care with non-specific symptoms which may represent unrecognised episodes of hypoglycaemia.

## 2 Method

We performed a cross-sectional survey in one primary care practice (list size: ∼11,000, ∼3300 > 65 years old) based in a small market town and with a large rural patient population. The practice’s Egton Medical Information Systems (EMIS) database was used to identify all patients aged 65 or over who were treated with insulin (n = 79), sulphonylureas (but not insulin) (n = 85), or metformin only (n = 121), and 50 age-matched non-diabetic patients.

One author (SH: a geriatrician) systematically reviewed patients’ consultation notes over a one year period (5/2/12-4/2/13), to identify any episodes of hypoglycaemia (defined below), or any “hypo clue consultations” – consultations with non-specific symptoms known to be associated with hypoglycaemia (see below), where no other obvious explanation or subsequent diagnosis was recorded. The records were reviewed sequentially using the practice’s internal computer number for each patient (essentially a random number). Review of the consultation records was performed independently of patient characteristics which were collected on a separate occasion: age, diabetes details, treatment, and glycated haemoglobin (HbA1c) blood test results.

### 2.1 Definition of hypoglycaemia

Hypoglycaemia episodes were defined as episodes having been directly confirmed by a doctor or nurse, paramedic or hospital (although the blood glucose was not always recorded).

### 2.2 Definition of “hypo clue” consultations

A “hypo clue consultation” was defined as one or more of the following symptoms recorded in the primary care records, without an obvious explanation or subsequent diagnosis documented – or documentation that hypoglycaemia had been considered. The symptoms (or synonyms) included were based on the work by Jaap et al. [14]: shivering, shaking, sweating, pounding heart/palpitations, lip tingling, dry mouth, apprehension, anxiety, agitation, confusion, odd behaviour, lethargy/fatigue, tiredness, drowsiness, weakness, speech difficulty, light-headedness, dizziness, unsteadiness, incoordination, feeling unwell, nausea, hunger, headache, double or blurred vision, depression symptoms, difficulty concentrating, and memory complaints [14]. Unexplained waking and falls were also included due to clinical experience.

### 2.3 Analysis

The majority of the data was non-parametric; thus median results and interquartile ranges are presented, and chi^2^/Fisher’s exact tests used for comparing frequencies across groups and for the binary analyses of “at least one” hypoglycaemia episode or “hypo clue” consultation over the year by treatment group.

Frequency of presentation with individual “hypo clue” symptoms was assessed. Individual symptom frequencies were compared in patients who had, and those who had not had, a recognised episode of hypoglycaemia on another occasion, using chi^2^/Fisher’s exact tests.

The median HbA1c of those with/without at least one hypoglycaemia or “hypo clue” consultation per treatment group was compared using the Mann Whitney test.

### 2.4 Ethics

The research project was based on an initial audit within the practice, which did not require ethical permission.

## 3 Results

### 3.1 Frequency of hypoglycaemia

**Fig. 1:**
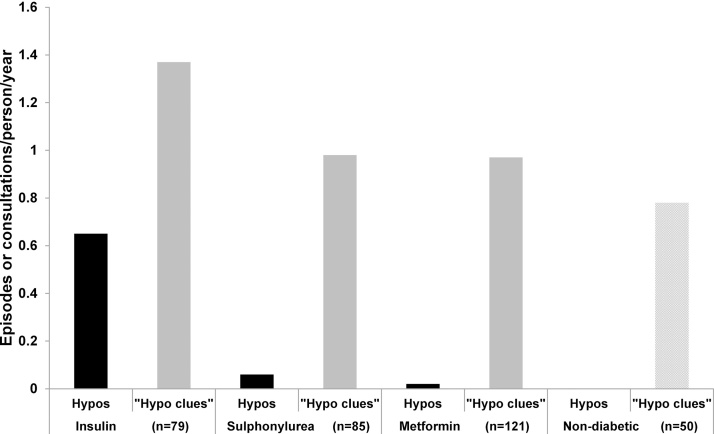
Frequency of documented hypoglycaemia and “hypo clue” consultations (per person per year) according to treatment group, in patients >65 years. p < 0.001 for a difference in rates of hypoglycaemia across the groups; p = 0.34 for a difference in rates of “hypo clue” consultations.

At least one episode of hypoglycaemia was recorded for 27/79 (34%) insulin-treated patients, compared to 4/85 (5%) sulphonylurea-treated patients, 2/121 (2%) metformin-only treated patients, and none in patients without diabetes. The total frequency was significantly higher in insulin-treated patients: 51 episodes (0.65 episodes/patient/year), compared to 5 episodes in the 85 patients with sulphonylureas (0.06 episodes/patient/year), and 3 in the 121 (0.02 episodes/patient/year) for the metformin-only treated patients, p < 0.001, Fig. 1.

### 3.2 Frequency of “hypo clue” consultations

Even patients without diabetes had frequent consultations with at least one non-specific symptom without other obvious documented explanation (feasibly due to hypoglycaemia in an at-risk patient), 0.78 consultations/patient/year (39 consultations in 50 patients). Rates of “hypo clue” consultations were similar for all patients with diabetes, regardless of treatment (insulin 1.37, sulphonylureas 0.98, metformin 0.97 consultations/patient/year; p = 0.34), Fig. 1.

### 3.3 Reported symptoms in “hypo clue” consultations

The most commonly reported non-specific symptoms overall in this study, in decreasing order of frequency, were lethargy/tiredness (47/335, 14%), falls (46/335, 13.7%), feeling unwell (37/335, 11%), dizziness/light-headedness (35/335, 10.5%), depression symptoms (28/335, 8.4%), nausea (21/335, 6.3%), and unsteadiness (18/335, 5.4%).

### 3.4 Consultation with possible “hypo clue” symptoms in those with/without documented hypoglycaemia

**Fig. 2:**
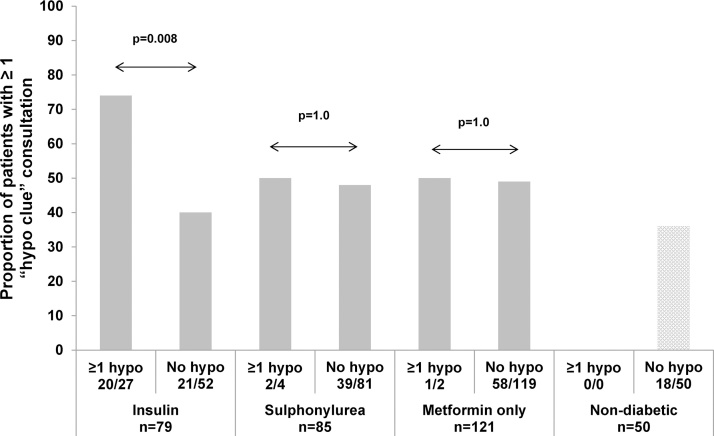
Proportion of patients who had at least one documented “hypo clue” consultation over the year, and whether they had also had a documented episode of hypoglycaemia over the year.

In those patients who were insulin-treated and had at least one documented episode of documented hypoglycaemia over the year, 20/27 (74%) had presented on at least one other occasion with a “hypo clue” symptom, Fig. 2. This was in comparison to 21/52 (40%) of those insulin-treated patients without a documented hypoglycaemia episode, p = 0.008. In sulphonylurea and metformin treated patients with at least one document episode of hypoglycaemia over the year, 2/4 (50%) and 1/2 (50%) respectively had also presented at least once with possible “hypo clue” symptoms, with no difference in rates between those with or without documented hypoglycaemia, p = 1.0. The odds ratio for insulin-treated patients having a hypoglycaemia episode if they had consulted on another occasion with a possible “hypo clue” symptom, was 4.2, compared to 1.1 in sulphonylurea or metformin only-treated patients.

### 3.5 Symptoms in “hypo clue” consultations in those with/without documented hypoglycaemia

When the rates were compared overall in those with/without at least one episode of documented hypoglycaemia, the symptoms that were significantly more common were nausea (7/33, 21.2% vs 14/302, 4.6%, p = 0.002), falls (10/33, 33.3% vs 36/302, 11.9%, p = 0.007), unsteadiness (5/33, 15.2% vs 13/302, 4.3%, p = 0.02), and depression symptoms (6/33, 18.2% vs 22/302, 7.3%, p = 0.044).

The majority of patients (27/33, 81.8%) with at least one documented episode of hypoglycaemia were insulin-treated. Of these, 9/27 (33.3%) had presented on another occasion with a fall, compared to 4/52 (7.7%) insulin-treated patients without a documented episode of hypoglycaemia, p = 0.008; and a higher proportion had presented with unsteadiness (5/27, 18.5% vs 2/52, 3.9%), p = 0.043. Presentation with nausea was also more frequent in those insulin-treated patients with a recognised/reported episode of hypoglycaemia over the year: 6/27 (22.2%) vs 1/52 (1.9%), p = 0.006.

### 3.6 Relationship with HbA1c

**Fig. 3:**
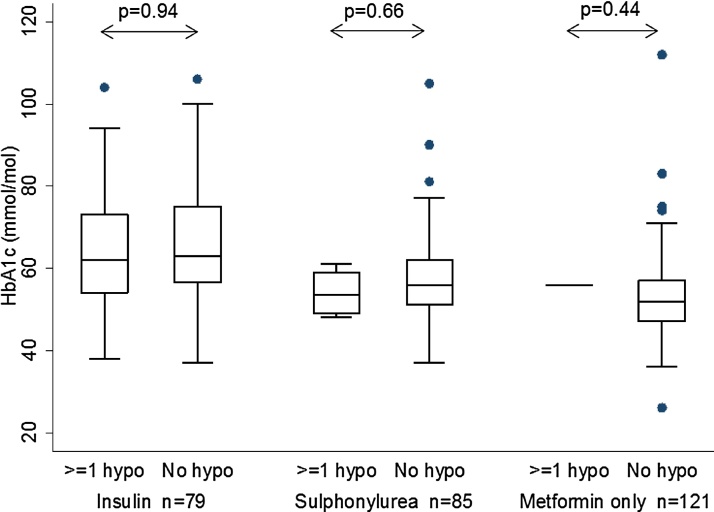
HbA1c in those patients with or without a documented episode of “definite” hypoglycaemia.

Hypoglycaemia was unrelated to HbA1c (p > 0.4), Fig. 3. There was also no relationship with “hypo clue” consultations and HbA1c, p > 0.3 for all.

## 4 Discussion

Non-specific symptoms are a common presentation to primary care in patients over 65 with and without diabetes. However we have shown that patients at high risk of hypoglycaemia – patients over the age of 65 who are insulin-treated and have had a recognised episode of hypoglycaemia, present to primary care on other occasions with unexplained non-specific symptoms which may represent unrecognised hypoglycaemia. Falls, unsteadiness and nausea are particularly worth noting.

### 4.1 Strengths and limitations

This study examined the difficult question of whether additional episodes of hypoglycaemia might be being missed in the older population. Primary care consultation records from just one primary care practice were used, albeit a reasonably large one. This meant reliance on documentation (coding and free text) by a limited number of staff; however this may also have offered more internal consistency for recording and comparing the vague symptoms between patients in this population. The similar rates of “hypo clue” consultations seen in sulphonylurea, metformin and non-diabetic patients is reasonably reassuring for consistency of the approach for identification of these consultations.

The “hypo clue” consultation definition used was deliberately all-embracing – hence high rates were seen in patients without diabetes. Even so, more “hypo clue” consultations were seen in insulin-treated patients who had also had a documented episode of hypoglycaemia. This rate may be artificially elevated as this group of patients could consult more often. We tried to address this by presenting the results comparing those with and without episodes of hypoglycaemia within treatment groups.

The definition of hypoglycaemia, in contrast, was taken as a strict definition, i.e. only those episodes documented as having been confirmed in some way by a healthcare professional. This will under-estimate the frequency of hypoglycaemia, and may preferentially identify more “severe” episodes. This approach combined with the small study size may have limited the chance of identifying possible associations with those patients experiencing hypoglycaemia. In order to corroborate any relationships seen, a prospective study of possible “hypo clue” symptoms and hypoglycaemia could be undertaken.

Although not captured in the current analysis, less “severe” episodes of hypoglycaemia (e.g. those which were self-treated and not reported to primary care) are obviously also important: they may potentially pose a risk for development of reduced hypoglycaemia awareness and a subsequent more “severe” event, in addition to as yet under-appreciated possible effects e.g. on long-term cognition. A study which also directly asked patients about their experience/frequency of hypoglycaemia may prove valuable, perhaps combined with a more intense but objective assessment of hypoglycaemia, e.g. using continuous glucose monitoring.

Further study in a bigger dataset could be revealing: e.g. an “index” event of hypoglycaemia taken and preceding consultations analysed to see if “hypo clue” consultations preceded a recognised event – and thus potentially expose more robust “red flag” symptoms – or corroborate those suggested in the current study. A larger study would also allow more sophisticated analyses to be done, in particular corrections for factors which may have an impact on risk, such as age [30], [31], comorbidities [31], [32], and renal function [30]. In addition, insulin-treated patients in the current study comprise a heterogeneous group – i.e. some with long-standing Type 1 diabetes, and others with Type 2 diabetes and more recent initiation of insulin treatment. Although these patients may have different rates of presentation with hypoglycaemia or “hypo clues”, the underlying type of diabetes in clinical care is not always clearly defined or obvious [33], and thus an all-encompassing “insulin-treated” group was felt to be a more useful analysis in the current study.

Finally, HbA1c analysis was limited as it was based on a single HbA1c level from the year; and therefore does not reflect potential variation (and possible altered risks) over the year. No apparent relationship with hypoglycaemia or “hypo clues” was seen in the current study. This particular practice had been subject to a similar audit previously, and thus it is possible that the frequency of patients with very low HbA1c was lower than average.

### 4.2 Comparison with previous literature

Consistent with published literature, we found that documented hypoglycaemia is more frequent in insulin-treated patients, and the finding that 34% of insulin-treated patients had a “definite” episode of hypoglycaemia confirmed by a healthcare professional over the year is consistent with the 7–46% in insulin-treated patients of different durations in the UK Hypoglycaemia Study [6]: as previously mentioned, the insulin-treated patients in the current study comprised a heterogeneous group. 5% of sulphonylurea-treated patients having an episode of hypoglycaemia is also consistent with the 7% seen in the UK Hypoglycaemia Study [6].

18/50 (36%) of patients without diabetes had a “hypo clue” consultation by our definition. As previously discussed, this was an all-embracing definition, which included many non-specific symptoms frequently presenting to primary care. Although not directly comparable, other primary care studies have found 22-48% patients presenting with symptoms which could not be given a same-day diagnosis [23].

Regarding presentation with non-specific symptoms, lethargy/fatigue, feeling “generally unwell”, falls, and light-headedness/dizziness were the most frequently reported, each in over 10% of these patients aged >65. However falls and unsteadiness, along with nausea, were reported significantly more frequently in those who had also had (on another occasion) a hypoglycaemia episode. Overall 21% of those with at least one episode of documented hypoglycaemia over the year had attended on another occasion with nausea without a documented diagnosis, in comparison to 5% of those without an episode of hypoglycaemia – and 22% vs 2% in those who were insulin-treated.

It is perhaps not surprising falls were one of the most frequently presenting symptoms, as they are one of the most dramatic. However the difference of 30% vs 12% (or 33% vs 8% of insulin-treated) patients presenting with a fall in those who had/had not also had a documented hypoglycaemic episode on a different occasion is marked. Kachroo et al. [34] identified in a study of over 21,000 patients with Type 2 diabetes, those aged >75 who had experienced a documented episode of hypoglycaemia over a one-year period had an adjusted odds ratio (aOR) for a fall-related event of 1.77 (95% CI 1.48–2.12), and an increased risk was seen in patients with recurrent episodes of hypoglycaemia.

Recognised symptoms associated with hypoglycaemia which could predispose to falls include shakiness, anxiety, confusion, lethargy/fatigue, tiredness, drowsiness, weakness, light-headedness, dizziness, unsteadiness, incoordination, and double or blurred vision [35]. The finding that unsteadiness was the other most notable discriminatory symptom may be consistent with this: 15% vs 4% (or 19% vs 4% of insulin-treated) patients with/without hypoglycaemia on another occasion presented with unsteadiness. When originally reviewing the symptoms associated with hypoglycaemia in the elderly in comparison to younger adults, Jaap et al. identified that unsteadiness and light-headedness were amongst the most frequently occurring and intense [14]. This study was done by asking 102 insulin-treated patients with Type 2 diabetes who had experienced hypoglycaemia in the preceding 2 months their subjective experience of the presence of 22 symptoms of hypoglycaemia during a ‘typical’ hypoglycaemic episode. Falls were not given as an option in this study, and interestingly nausea had a low frequency (6%).

In contrast to the current study, a large meta-analysis showed a 30% increase in severe hypoglycaemia with tight glycaemic control in people with Type 2 diabetes [4]. The apparent lack of relationship in the current study may reflect the low rates of “definite” hypoglycaemia documented, and at the other end of the spectrum, the all-embracing definition for “hypo clue” consultations used in this relatively small study may have masked results.

### 4.3 Clinical implications

Patients >65 and who are insulin-treated are at the highest risk of hypoglycaemia documented in primary care records, as will be well-recognised by primary care practitioners. However, hypoglycaemia in older adults is associated with non-specific and less intense symptoms than in younger people [13], [14], [15]. It is known to be under-reported to healthcare professionals [18], [36], which can be due to a failure to appreciate its significance, or poor recognition [16], [17] perhaps particularly in those with Type 2 diabetes, who may not have had education to go with the increased risk of hypoglycaemia with insulin [6], or with increasing duration of diabetes [9]. Symptoms can differ between episodes in the same person, which can make recognition especially challenging [19]. Additionally episodes of hypoglycaemia can be poorly recalled by patients [37], [38], which may be exacerbated by cognitive impairment. There may also be a fear of its implications such as relating to driving [36]. However, as previously discussed, it carries a high morbidity [8]. This means healthcare professionals need to take a more pro-active approach in enquiring about hypoglycaemia.

This study suggests those who have had a recognised episode of hypoglycaemia seem more likely to present on another occasion with a non-specific symptom which could conceivably be due to hypoglycaemia, and nausea, falls and unsteadiness seem to be particularly notable. The likelihood of this is corroborated by other published data, and as such, insulin-treated patients presenting with these symptoms should be reviewed with hypoglycaemia in mind.

More recent guidelines for older adults [10], [11], [12] favour a more common-sense approach in actively addressing glycaemic targets, particularly in a more elderly and frail population, who can ill-afford to be exposed to risk factors for accidents [35], [39], [40] and cognitive decline [41], [42], [43]. As previously discussed the current study did not show a clear relationship between hypoglycaemia or “hypo clue” consultations and HbA1c, but much larger meta-analyses have [44] – and additionally increased all-cause mortality has been observed with HbA1c results below 7.5% [45]. On the other hand, avoidance of hypoglycaemia is not as simple as relaxing HbA1c targets – Munshi et al [46] demonstrated using continuous glucose monitoring that 65% of a group of (mainly insulin-treated) elderly patients with HbA1c >8% experienced at least one episode of hypoglycaemia (blood glucose <3.9 mmol/L) over 3 days’ monitoring.

At the very least however, clinicians should be alert to the possibility of unrecognised hypoglycaemia in their older insulin-treated patients, and review them with this in mind.

## 5 Conclusion

Non-specific symptoms which can be symptoms of hypoglycaemia are common in a population over 65. However in insulin-treated patients at risk of hypoglycaemia, these “hypo clue” symptoms, in particular nausea, falls and unsteadiness, may represent episodes of hypoglycaemia not recognised by the patient. Thus GPs should consider a review, including of diabetes medication, when patients report or present with these symptoms.

## Conflict of interest

The authors state that they have no conflict of interest.

## Funding sources

ATH is an NIHR and a Wellcome Trust Senior Investigator.
